# Associations between serum leptin levels, hyperlipidemia, and cholelithiasis in dogs

**DOI:** 10.1371/journal.pone.0187315

**Published:** 2017-10-31

**Authors:** Sungin Lee, Oh-kyeong Kweon, Wan Hee Kim

**Affiliations:** Department of Veterinary Clinical Sciences, College of Veterinary Medicine and Research Institute for Veterinary Science, Seoul National University, Seoul, Republic of Korea; Universita degli Studi di Napoli Federico II, ITALY

## Abstract

Leptin and its receptor play several physiological roles in the canine gallbladder, and the dysregulation of leptin might play a role in the pathogenesis of gallbladder diseases such as gallbladder mucocele. Previous studies revealed a positive association between hyperlipidemia and gallstones in humans. However, the latter is still unclear in dogs with cholelithiasis. In this study, we examined the differences in leptin, leptin receptor, total cholesterol, and triglyceride levels between healthy dogs and dogs with cholelithiasis, and evaluated the correlation between leptin and hyperlipidemia. Twenty-eight healthy dogs and 34 client-owned dogs with cholelithiasis were enrolled in the study. Leptin concentrations and lipid profiles were determined from sera, and leptin and leptin receptor expression levels were quantified in gallbladder tissue. In dogs with cholelithiasis, serum concentrations of leptin (*p* < 0.001), total cholesterol (*p* < 0.001), and triglycerides (*p* < 0.001) were significantly higher compared with those in healthy dogs. Positive correlations were observed between serum leptin and total cholesterol (95% confidence interval (CI) = 0.61–0.89, *r* = 0.725, *p* < 0.001), and between leptin and triglycerides (95% CI = 0.63–0.89, *r* = 0.782, *p* < 0.001) in the cholelithiasis group. Hypercholesterolemia (Odds Ratio (OR) = 9.720; 95% CI = 1.148–82.318) and hypertriglyceridemia (OR = 12.913; 95% CI = 1.548–107.722) were shown to be risk factors for gallstone disease. In cholelithiasis patients who underwent cholecystectomy, serum leptin levels were significantly higher than in patients that had not undergone surgery (*p* < 0.001). Leptin and leptin receptor expression was upregulated in the gallbladder tissues of cholelithiasis patients (*p* < 0.01 and *p* < 0.001, respectively). These results indicate that increased serum leptin concentrations and hyperlipidemia (hypercholesterolemia or hypertriglyceridemia) are associated with canine cholelithiasis and that homeostatic imbalance of these parameters might affect the pathogenesis of gallstones.

## Introduction

Cholelithiasis is a pathological condition in which choleliths are found within the gallbladder or intrahepatic or extrahepatic ductal system [1]. Most dogs with cholelithiasis are asymptomatic, and choleliths are discovered incidentally during diagnostic imaging procedures or postmortem examination. However, cholelithiasis is one of the major causes of a life-threatening biliary tract disease, such as extrahepatic biliary tract obstruction caused by the migration of one or more choleliths into the common bile duct, and it may also be related to the occurrence of cholecystitis and pancreatitis [2, 3]. Whereas dietary-induced cholesterol gallstones are common in humans, canine choleliths are primarily composed of calcium bilirubinate with cholesterol and bilirubin [1, 2]. Although the exact etiopathogenesis of cholelithiasis in dogs is unclear, based on studies in humans and experimental animals, it has been suggested that decreased gallbladder motility, excess secretion of mucin, and altered absorption/secretion of the gallbladder are involved in the pathogenesis [4–8].

Leptin is a protein hormone that is involved in the regulation of energy homeostasis and food intake at the level of hypothalamus, and it is mainly produced in mature adipocytes [9]. During the past 20 years since its discovery, a tremendous amount of knowledge pertaining to leptin has emerged, linking it to fat metabolism and nutrition and suggesting its role as a pathophysiological modulator in various diseases, such as endocrinopathy [10, 11], heart disease [12], and tumors [13, 14]. The expression of leptin and its receptors was investigated in different organs, such as small intestine [15], salivary glands [16], and pancreas [17], which showed that leptin may represent a physiological regulatory factor in these organs.

The results of several studies suggested a relationship between leptin and gallbladder. In our previous study, by investigating the distribution of leptin and its receptor, we confirmed that the canine gallbladder is not only a source of leptin, but it is also affected by it [18]. Based on these results, we have subsequently evaluated the differences in circulating leptin concentrations, and leptin and its receptor expression in tissue samples of dogs with gallbladder mucocele (GBM). The results of that study indicated that the impairment of leptin expression may be associated with the pathogenesis of GBM and that leptin concentrations may correlate with the severity of GBM [19]. Moreover, previous studies showed that leptin represents a key factor during gallstone formation, either directly or indirectly, by modulating gallbladder motility and regulating the expression of gallbladder genes related to the secretion and reabsorption of electrolytes and water [8, 20]. Another study in humans showed that the serum leptin concentrations in patients with cholelithiasis were higher than those in controls [21].

Although previous studies performed in both humans and animals revealed multiple roles of leptin in various organs, little information is available regarding the correlation between leptin and cholelithiasis in dogs. Investigations on the relationship between leptin and hyperlipidemia [22, 23] and between hyperlipidemia and gallstones [24] showed some indications of the associations between them, but they remain still unclear. Therefore, the objective of this study was to evaluate possible links between leptin, hyperlipidemia, and the development of cholelithiasis in dogs. We assessed circulating leptin concentrations and lipid profiles, such as total cholesterol and triglyceride levels, using enzyme-linked immunosorbent assay (ELISA) and an automated biochemical analyzer, respectively. In addition, tissue concentrations of leptin and its receptor were measured in the gallbladder using real-time polymerase chain reaction (PCR) in dogs with cholelithiasis and healthy dogs.

## Materials and methods

### Sample preparation

In this study, 34 client-owned dogs diagnosed with cholelithiasis and 28 healthy control dogs were recruited from the Veterinary Medical Teaching Hospital at Seoul National University (SNU). Additionally, the control group included 10 healthy laboratory beagle dogs housed at the Department of Veterinary Surgery, College of Veterinary Medicine at SNU for gallbladder sampling. The body condition score (BCS) of each dog enrolled this study was evaluated by one investigator using a 9-point scaling system [25]. All dogs with a normal BCS (5-6/9) were selected and underwent physical examination, imaging examinations such as X-ray and abdominal ultrasonography, complete blood counts, and serum biochemical analysis. All gallbladders of the control group were confirmed to be normal based on abdominal ultrasonography and blood analyses. Dogs with cholelithiasis were diagnosed using abdominal X-ray and ultrasonography at the Department of Veterinary Radiology, College of Veterinary Medicine, SNU. Diabetes mellitus (DM) was diagnosed based on the presence of appropriate clinical signs, persistent fasting hyperglycemia (> 200 mg/dL), and concomitant glycosuria [26, 27]. DM patients showed the classical clinical signs of polyuria, polydipsia, polyphagia, and weight loss [27]. Insulin resistance was determined by persistent observation of blood glucose levels over 200 mg/dL when measured every 2 h over 12 h period in diabetic patients treated with an insulin dose greater than 1.0 U/kg [28].

For biochemical analysis, jugular venipuncture was performed in 34 dogs diagnosed with cholelithiasis, 18 control client-owned healthy dogs and 10 control laboratory beagle healthy dogs. Food was withheld from the animals 12 h prior to venipuncture. Blood was collected in the serum separation tubes and centrifuged for 10 min at 1000 ×*g*. One portion of serum sample was immediately used for the determination of triglyceride, total cholesterol, and glucose levels, and the remainder was frozen at −80°C until further ELISA analyses and insulin concentration measurements. Total serum cholesterol, triglyceride, and glucose levels were measured spectrophotometrically using a Hitachi 7180 automated biochemical analyzer (Hitachi, Tokyo, Japan) with commercial kits (JW Pharmaceutical, Seoul, Republic of Korea), and serum insulin levels were measured using radioimmunoassay (HI-14K, Millipore, Billerica, MA, USA) at IDEXX Laboratories, Inc. (Westbrook, ME, USA). For the quantification of leptin and leptin receptor expression, gallbladder samples were obtained from 10 dogs diagnosed with cholelithiasis that underwent therapeutic cholecystectomy and 10 healthy control beagle dogs that were euthanized because of other reasons (SNU-160720-13) not related to this study. Dogs with cholelithiasis underwent surgical interventions when clinical symptoms, abnormalities in serum biochemistry and results of imaging tests associated with biliary obstruction were observed, or when gallbladder rupture was suspected. Gallbladder samples were stored at −80°C until their use in real-time PCR analysis. Informed consents were obtained from the owners, and all procedures were approved by the Seoul National University Institutional Animal Care and Use Committees (SNU-170220-1).

### Sandwich ELISA

Serum leptin concentrations in all samples were analyzed in duplicate using a commercial canine-specific leptin sandwich ELISA kit (Canine Leptin ELISA, Millipore, Billerica, MA, USA) according to the manufacturer’s protocol. The intra- and inter-assay coefficients of variation were 5% and 7%, respectively. The absorbances were determined using an automated microplate spectrophotometer (Epoch, BioTek Instruments Inc., Winooski, VT, USA) at 450 nm.

### Real-time PCR

Total RNA extraction was performed using a Hybrid-R RNA extraction kit (GeneAll Biotechnology, Seoul, Republic of Korea) according to manufacturer’s protocol, and RNA levels were determined using an automated microplate spectrophotometer (Epoch, BioTek Instruments Inc., Winooski, VT, USA). cDNA was synthesized using the RNA to cDNA EcoDry^™^ Premix (Clontech Laboratories Inc., Mountain View, CA, USA) with 1000 ng of extracted total RNA as a template, and the newly synthesized cDNA was amplified using real-time PCR in triplicate with a SYBR^®^ Premix Ex Taq^™^ II (Takara Bio Inc., Otsu, Japan) and StepOnePlus^™^ Real-Time PCR System (Applied Biosystems, Foster City, CA, USA) according to the manufacturer’s instructions. The level of a housekeeping gene, glyceraldehyde 3-phosphate dehydrogenase (*GAPDH*), was used for the normalization of leptin and leptin receptor expression levels. Primers for leptin and the leptin receptor in the canine gallbladder were reported previously [18], and primer sequences are shown in Table 1.

**Table 1:** Specific primer sequences for real-time polymerase chain reaction with amplicon sizes and optimal annealing temperatures.

Target gene^a^	Accession number	Primer sequence (5′ to 3′)		Amplicon size (bp)	Annealing temp. (°C)
		Forward	Reverse		
*GAPDH*	NM_001003142.2	CATTGCCCTCAATGACCACT	TCCTTGGAGGCCATGTGGAC	105	58
*Ob*	NM_001003070.1	ACCGTATGGGTGTCCTTTATCCT	AGAGTGGCTCTGTGGTGTGAGA	63	58
*Ob-R*	NM_001024634.1	CTTTTGCCTGCTGGAATCTC	TTGCTCCAAAAGCAACAGTG	143	58

^a^*GAPDH*, glyceraldehyde-3-phosphate dehydrogenase; *Ob*, leptin; *Ob-R*, leptin receptor.

### Statistical analysis

Statistical analyses were performed using SPSS, version 23.0 (SPSS Inc., Chicago, IL, USA). The Shapiro-Wilk method was used to evaluate the normal distribution of the obtained data. The Mann-Whitney *U*-test was performed in order to analyze differences in age, body weight, BCS, and biochemical characteristics such as serum leptin, total cholesterol, triglyceride, glucose, and insulin concentrations between the control and cholelithiasis groups. The Kruskal-Wallis test was used for comparisons of serum leptin concentrations and serum insulin levels between three groups (healthy dogs, cholelithiasis patients that did not undergo a surgery, and cholelithiasis patients that underwent surgery; or healthy dogs, cholelithiasis patients without DM, and cholelithiasis patients with DM). When a significant difference was obtained, post-hoc comparisons were performed using the Mann-Whitney test with Bonferroni correction. The data are presented as the median value of each group followed by the interquartile range. The independent *t*-test was used to compare the total cholesterol and triglyceride levels between dogs with cholelithiasis that did not undergo surgery and dogs with cholelithiasis that underwent surgery, and to analyze the relative mRNA expression levels of leptin and its receptor in gallbladder samples in the healthy and patient groups. These data are presented as mean and standard deviation. The relationship between the serum leptin concentration and total cholesterol, triglycerides, glucose, insulin, age, sex, breed, body weight, and BCS was estimated using the Spearman rank correlation test. The univariate odds ratio (OR) and 95% confidence interval (CI) for cholelithiasis associated with hypercholesterolemia, hypertriglyceridemia, hyperglycemia, or hyperinsulinemia were evaluated using Fisher’s exact test. A *p*-value < 0.05 was considered statistically significant.

## Results

### Cases

Detailed basic information and the results of blood analyses of 28 healthy control dogs and 34 dogs diagnosed with cholelithiasis are summarized in Table 2. The median age of the dogs with cholelithiasis was 12.5 years, which was significantly higher than that of the healthy control dogs (8 years old) (*p* < 0.001). Body weight and BCS did not significantly differ between the groups (*p* = 0.218 and *p* = 0.403, respectively). Seven of 34 dogs with cholelithiasis (20.6%) were found to have DM, and two of them showed insulin resistance. Of seven diabetic dogs, one had hypercholesterolemia, while two had hypertriglyceridemia.

**Table 2:** Comparison of age, sex, breed, body weight (BW), body condition score (BCS), diabetes mellitus (DM), and biochemical characteristics between healthy dogs (Controls) and dogs with cholelithiasis.

	Healthy Dogs (n = 28)	Dogs with Cholelithiasis (n = 34)
Age (years)^a^	8.00 (3.00)	12.50 (3.25) ***
Sex (n)	Males (7) Castrated males (9) Females (6) Spayed females (6)	Castrated males (15) Females (3) Spayed females (16)
Breed (n)	Beagle (10) Mixed breed (4) Poodle (3) Maltese (2) Shih Tzu (2) Chihuahua (2) Spitz (2) Bichon Frise (2) Yorkshire Terrier (1)	Maltese (7) Poodle (5) Schnauzer (4) Cocker Spaniel (4) Shih Tzu (3) Mixed breed (3) Welsh Corgis (2) Pekingese (2) Yorkshire Terrier (2) Golden Retriever (1) Spitz (1)
BW (kg)	7.20 (3.87)	5.44 (4.94) ^NS^
BCS (out of 9)^b^	5 (5–6)	5 (5–6) ^NS^
Diabetes mellitus (n)	—	7
Total cholesterol (mg/dL)	151.00(58.00)	270.00 (106.25) ***
Triglycerides (mg/dL)	58.00 (41.25)	117.00 (99.75) ***
Glucose (mg/dL)	110.05 (18.25)	106.50 (50.25) ^NS^
Insulin (μIU/mL)	6.75 (10.75)	14.15 (21.18) *

^a^ Continuous variables are presented as median values followed by interquartile range.

^b^ BCS, which is a discontinuous variable, is presented as the median followed by range.

Reference ranges are as follows: Total cholesterol [112–312]; triglycerides [21–133]; glucose [75–120]; and insulin [5.2–41.5].

*p < 0.05,

***p < 0.001,

^NS^*p* > 0.05 compared with controls.

The gallbladder tissue samples used in this study were acquired from 10 healthy beagle dogs registered as experimental animals and 10 patients that underwent cholecystectomy. Information regarding these dogs is summarized in Table 3. The median age was significantly higher in the cholelithiasis group (14 years old) than in the healthy control group (8 years old) (*p* < 0.001). Mean body weight was significantly higher in the healthy control group (8.31 kg) than in the cholelithiasis group (5.21 kg) (*p* < 0.001). The median BCS of the healthy control group was 5/9, which was similar to that of the cholelithiasis group (*p* = 0.661). One of the 10 dogs with cholelithiasis (10%) that underwent surgery had DM, but insulin resistance was not observed.

**Table 3:** Comparison of age, sex, breed, body weight (BW), body condition score (BCS), diabetes mellitus (DM), and biochemical characteristics between healthy dogs (Controls) and dogs with cholelithiasis subjected to cholecystectomy.

	Healthy Dogs (n = 10)	Dogs with Cholelithiasis (n = 10)
Age (years)^a^	8.00 (1.25)	14.00 (4.00) ***
Sex	Males (2) Castrated males (3) Females (3) Spayed females (2)	Castrated males (4) Females (1) Spayed females (5)
Breed (n)	Beagle (10)	Maltese (3) Schnauzer (2) Poodle (2) Shih Tzu (2) Cocker Spaniel (1)
BW (kg)	8.31 (0.62)	5.21 (1.83) ***
BCS (out of 9)^b^	5 (5–6)	5 (5–6) ^NS^
Diabetes mellitus (n)	—	1
Total cholesterol (mg/dL)	158.70 (42.03)	300.40 (129.73) **
Triglycerides (mg/dL)	50.00 (29.75)	92.50 (107.50) **
Glucose (mg/dL)	107.00 (32.25)	110.50 (31.75) ^NS^
Insulin (μIU/mL)	6.30 (12.27)	18.60 (29.08) ^NS^

^a^ Continuous variables are presented as means followed by SD, except for age, triglycerides, glucose, and insulin, which are presented as the median followed by interquartile range.

^b^ BCS, which is a discontinuous variable, is presented as the median followed by range.

Reference ranges are as follows: Total cholesterol [112–312]; triglycerides [21–133]; glucose [75–120]; and insulin [5.2–41.5].

**p < 0.01,

***p < 0.001,

^NS^*p* > 0.05 compared with controls.

### Analysis of serum leptin, total cholesterol, triglyceride, glucose, and insulin levels in healthy control dogs and dogs with cholelithiasis

**Fig 1:**
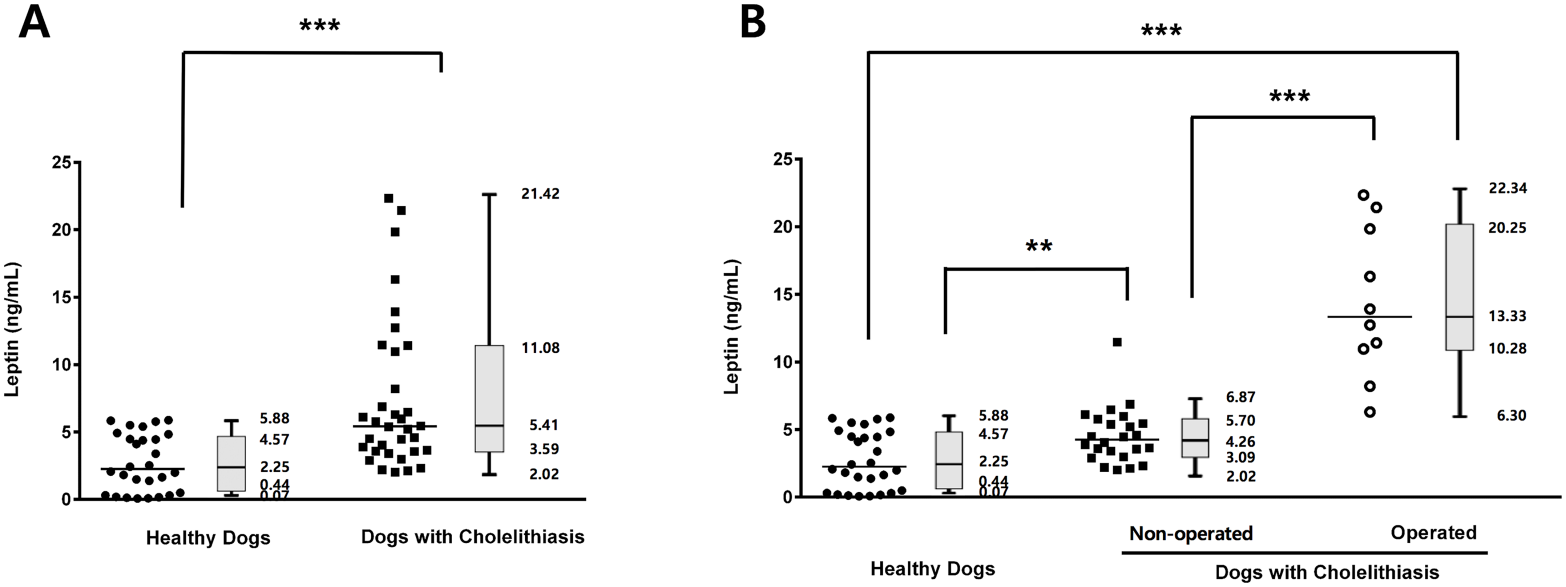
Circulating leptin levels. (A) In dogs with cholelithiasis (n = 34), serum leptin levels were significantly higher than those determined in healthy dogs (n = 28). (B) Serum leptin levels were significantly higher in cholelithiasis patients that underwent cholecystectomy compared with those in patients that did not undergo surgery. Horizontal bars in scatter plots indicate median values. **p < 0.01, ***p < 0.001 between groups.

**Fig 2:**
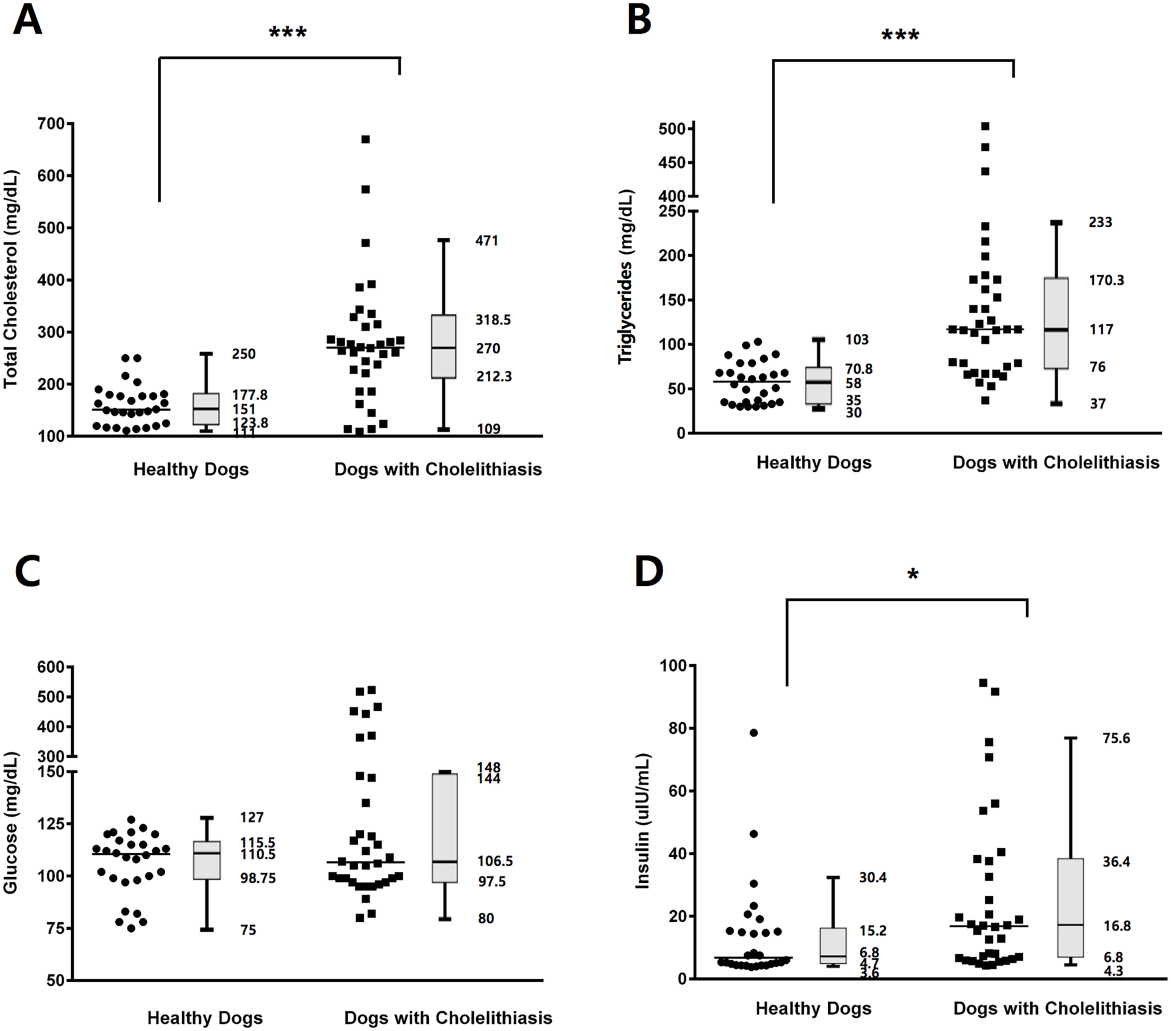
The results of biochemical and hormonal analyses. Serum (A) total cholesterol, (B) triglycerides, and (D) insulin concentrations were significantly higher in dogs with cholelithiasis (n = 34) compared with those in the healthy dogs (n = 28), but (C) glucose levels were not significantly different between the two groups. Horizontal bars in scatter plots indicate median values. *p < 0.05, ***p < 0.001 between groups.

**Fig 3:**
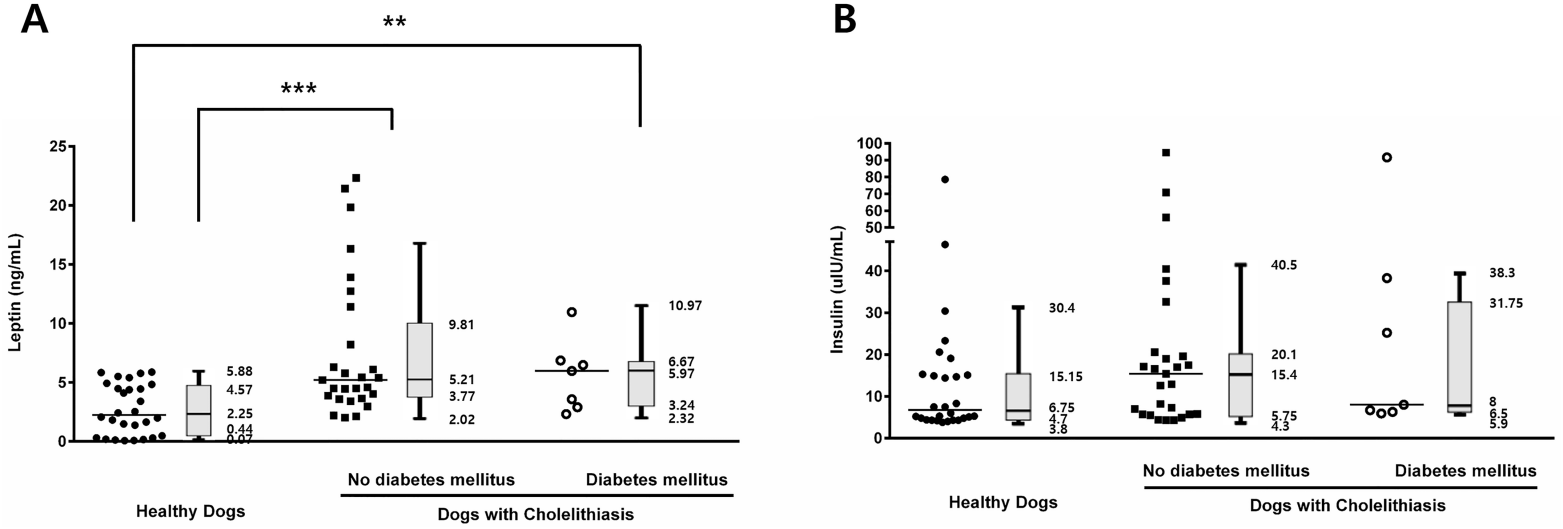
Serum leptin and insulin levels. The cholelithiasis group (n = 34) was divided according to the presence (n = 7) or absence (n = 27) of diabetes mellitus; the concurrence of diabetes with cholelithiasis had no significant effect on serum (A) leptin or (B) insulin levels. Horizontal bars in scatter plots indicate median values. **p < 0.01, ***p < 0.001 between groups.

In the cholelithiasis group, serum leptin concentrations were significantly higher than those in the healthy group (*p* < 0.001; Fig 1). To determine the changes in serum leptin concentration according to the severity of cholelithiasis, the cholelithiasis group was further categorized into dogs that underwent cholecystectomy and dogs that did not; serum leptin levels were significantly higher in dogs that underwent cholecystectomy (*p* < 0.001; Fig 1). The serum concentrations of total cholesterol (*p* < 0.001), triglycerides (*p* < 0.001), and insulin (*p* = 0.030) were significantly higher in dogs with cholelithiasis compared with those in healthy dogs, but glucose levels did not significantly differ between controls and patients (*p* = 0.524) (Fig 2). To evaluate the differences in leptin and insulin concentrations in the cholelithiasis patients with or without DM, the members of the cholelithiasis group were separated into dogs with DM and without it, and we showed that the concurrence of DM with cholelithiasis did not significantly affect serum leptin and insulin levels (*p* = 0.717 and *p* = 0.089, respectively; Fig 3).

### Relative expression levels of leptin and leptin receptor in healthy dogs and dogs with cholelithiasis

**Fig 4:**
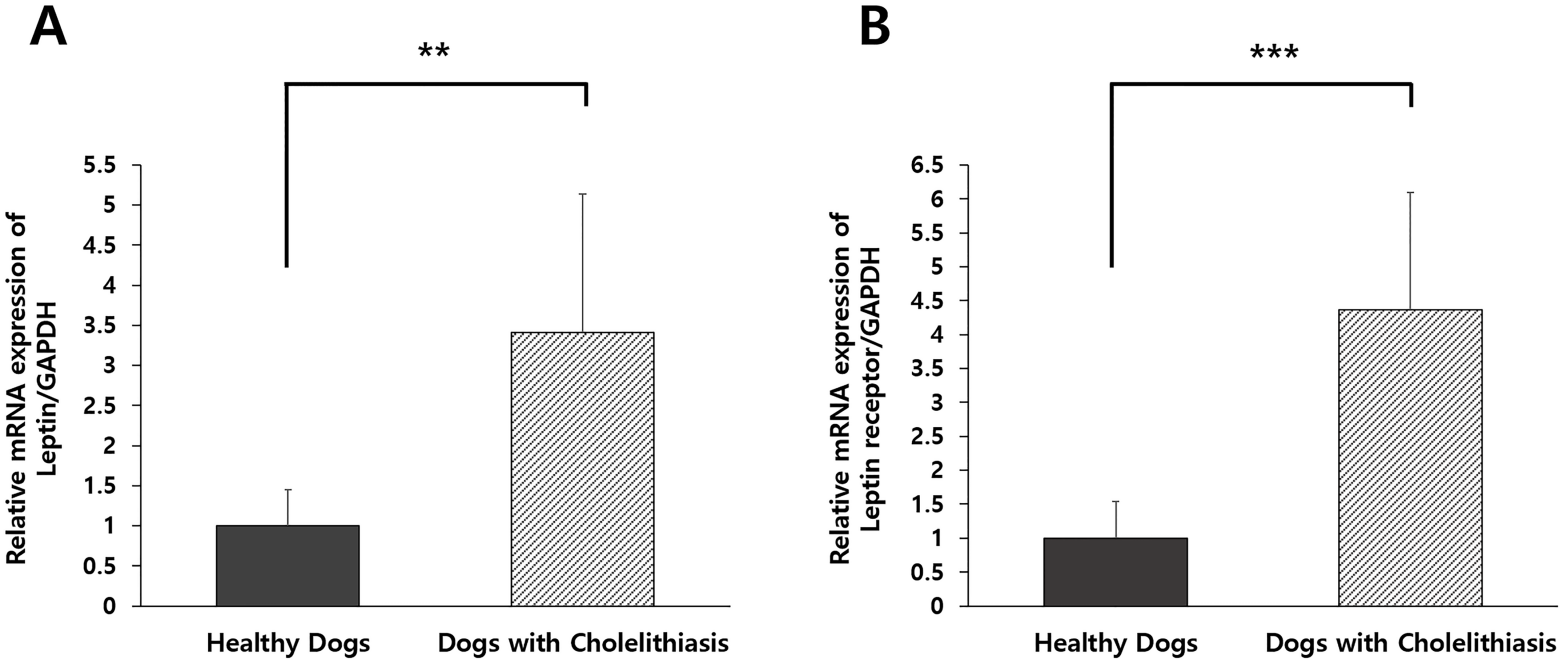
Relative mRNA expression levels of leptin and leptin receptor in gallbladder tissues. The mRNA expression levels of (A) leptin and (B) leptin receptor were significantly higher in patients with cholelithiasis (n = 10) than in the controls (n = 10). Columns indicate mean values, and vertical error bars represent standard deviation. **p < 0.01, ***p < 0.001 between groups.

The mRNA expression levels of leptin and its receptor were significantly upregulated in dogs with cholelithiasis, compared with those in the healthy control dogs (*p* < 0.01 and *p* < 0.001, respectively; Fig 4).

### Correlations

**Fig 5:**
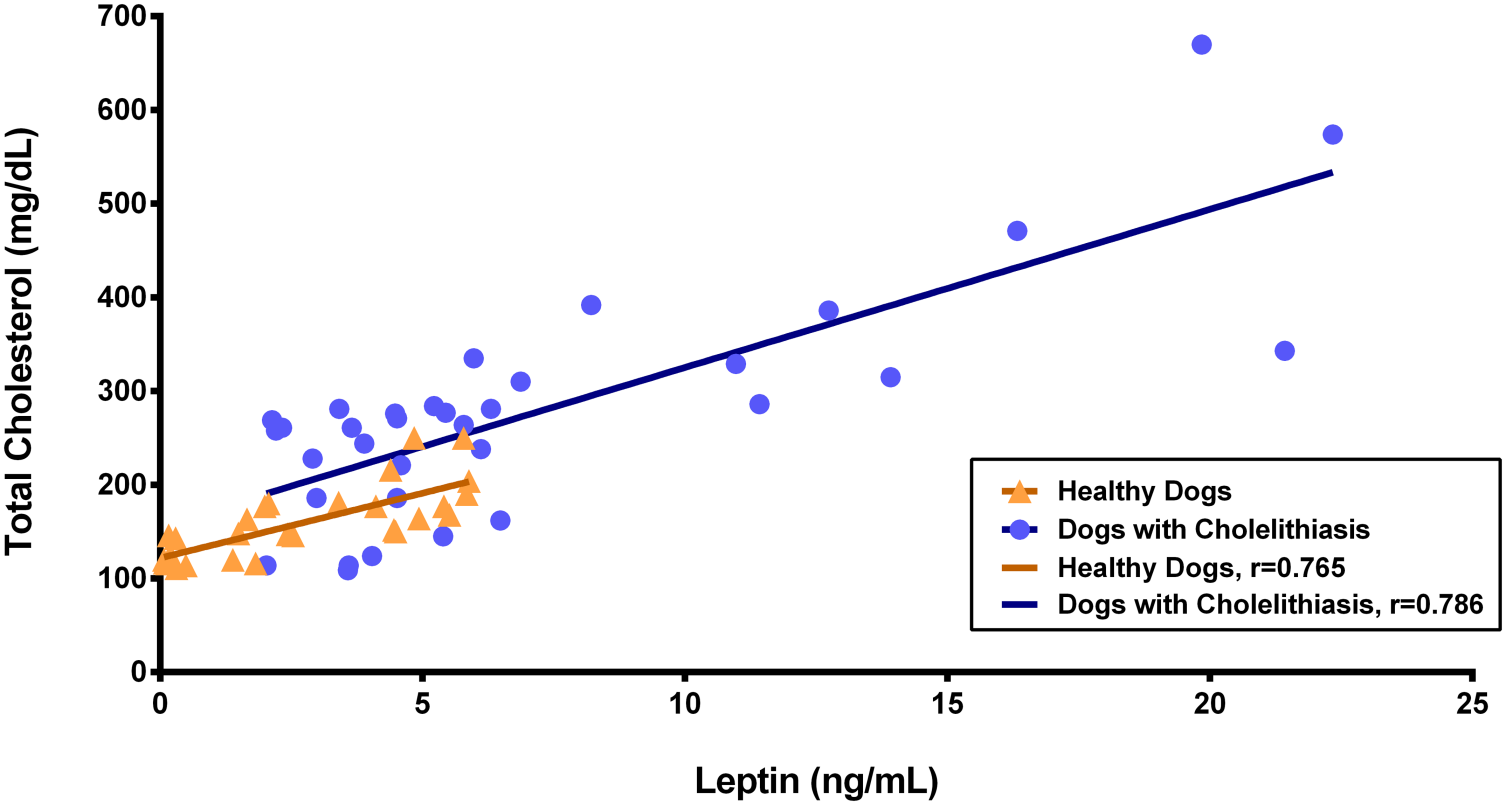
Correlation between serum leptin and total cholesterol levels. A significant positive correlation between serum leptin and total cholesterol levels was observed in both controls and patients.

**Fig 6:**
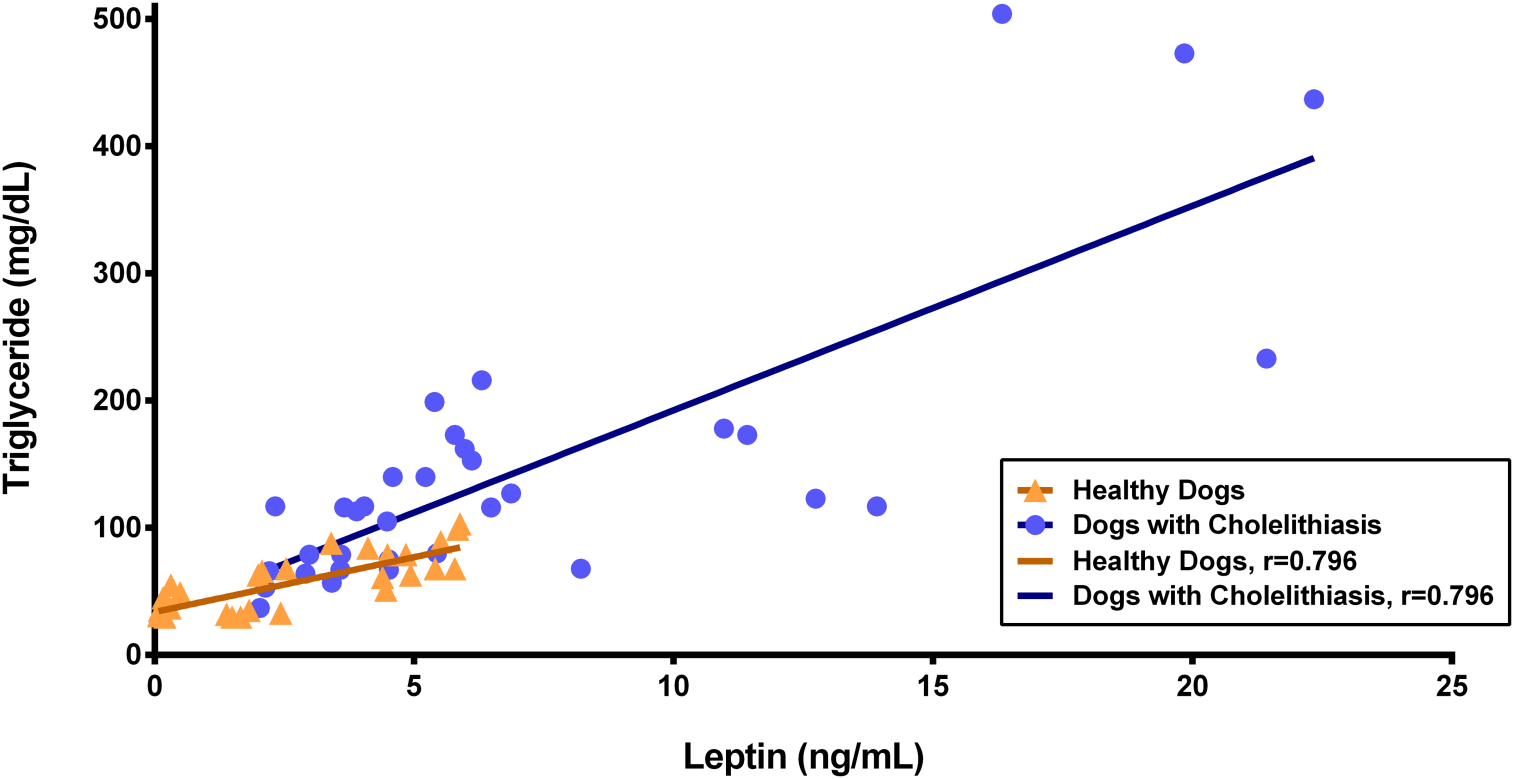
Correlation between serum leptin and triglyceride levels. A significant positive correlation between serum leptin and triglyceride levels was observed in both controls and patients.

A significant positive correlation was observed between serum leptin and total cholesterol in both control and patient groups (95% CI = 0.55–0.89, *r* = 0.794, *p* < 0.001 and 95% CI = 0.61–0.89, *r* = 0.725, *p* < 0.001, respectively; Fig 5). Additionally, significant positive correlations were observed between leptin and triglyceride levels in the control and patient groups (95% CI = 0.60–0.90, *r* = 0.788, *p* < 0.001 and 95% CI = 0.63–0.89, *r* = 0.782, *p* < 0.001, respectively; Fig 6). No correlations were detected between serum leptin levels and glucose (*p* = 0.215), insulin (*p* = 0.988), age (*p* = 0.171), sex (*p* = 0.708), breed (*p* = 0.498), body weight (*p* = 0.976), or BCS (*p* = 0.720) in healthy dogs or in patients with cholelithiasis (*p* = 0.931, 0.638, 0.740, 0.968, 0.777, 0.920 and 0.541, respectively). Fisher’s exact test showed that the OR of cholelithiasis in dogs with hypercholesterolemia (9/43 cases) was 9.72-fold higher than that of dogs without hypercholesterolemia (95% CI = 1.15–82.32, *p* = 0.017), and the OR of cholelithiasis in dogs with hypertriglyceridemia (11/34 cases) was 12.91-fold higher than that of dogs without hypertriglyceridemia (95% CI = 1.55–107.72, *p* = 0.008). There was no significant association between the presence of cholelithiasis and the presence of hyperglycemia (10/34 cases; OR = 1.92, 95% CI = 0.57–6.47, *p* = 0.377) or hyperinsulinemia (6/34 cases; OR = 2.79, 95% CI = 0.52–15.05, *p* = 0.276). Detailed information regarding the ORs obtained in dogs with cholelithiasis is shown in Table 4.

**Table 4:** Odds ratios obtained for the effects of hypercholesterolemia, hypertriglyceridemia, hyperglycemia, and hyperinsulinemia in dogs with cholelithiasis.

Factor	Number	Odds ratio	95% CI	*p*-value
Hypercholesterolemia	9/34	9.720 *	1.148–82.318	0.017
Hypertriglyceridemia	11/34	12.913 **	1.548–107.722	0.008
Hyperglycemia	10/34	1.917 ^NS^	0.568–6.468	0.377
Hyperinsulinemia	6/34	2.786 ^NS^	0.516–15.052	0.276

*p < 0.05,

**p < 0.01,

^NS^*p* > 0.05 compared with controls.

## Discussion

The results of this study showed that serum leptin concentrations were not significantly correlated with age, sex, breed, body weight, or BCS in either dogs with cholelithiasis or healthy dogs. In previous investigations, plasma leptin levels were significantly higher in dogs with increased BCS values; however, no significant effects of age, sex, or breed were found [29, 30]. These results confirm that plasma leptin levels may be a useful marker for evaluating adiposity regardless of variations in breed, age, or sex [29]. One study suggested that serum cholesterol, triglyceride, and glucose levels in dogs can be affected by the BCS increasing [31]. Based on previous studies suggesting that BCS is significantly associated with the circulating levels of leptin, cholesterol, triglycerides, and glucose [29–31], in order to rule out the influence of BCS on the measurement of these analytes, all examined dogs were selected based on similar BCS values (5-6/9).

Cholelithiasis was diagnosed based on the combination of biochemical analysis results, medical imaging, and clinical signs such as abdominal pain, vomiting, anorexia, and icterus. Biochemical analyses vary widely with cholelithiasis, and abdominal radiography is occasionally limited in its ability to confirm the presence of choleliths depending on the relative composition of calcium salt [1, 27]. Radiopaque gallstones account for 48% in dogs with cholelithiasis, which is higher than that in humans (15%). These results may be explained by a higher percentage of calcium bilirubinate-containing choleliths in dogs [2]. Abdominal ultrasonography is one of the most useful tools for the diagnosis of cholelithiasis. Ultrasonographically, choleliths are observed as echogenic focal structures, usually with acoustic shadowing within the gallbladder lumen, and might be accompanied by gallbladder wall thickening, dilation of the biliary tract, or pericholecystic fluid [2, 32]. However, even if these findings are observed via abdominal ultrasonography, they do not always correspond to the clinical symptoms and changes in laboratory findings. For this reason, in some cases, the use of ultrasonography is not sufficient to evaluate the prognosis of the disease. This study showed that the serum leptin concentrations in dogs with cholelithiasis were significantly higher than those in healthy dogs. To further estimate the association between serum leptin levels and the severity of cholelithiasis, dogs in the cholelithiasis group were divided into those that underwent cholecystectomy and those that did not, and leptin levels were found to be significantly increased in the operated group. These results are supported by previous studies suggesting that human patients with cholelithiasis exhibit higher serum leptin concentrations compared with those in the controls, and they indicate a relationship between leptin and the occurrence of gallstone disease [21, 33]. Therefore, our results further suggest that serum leptin levels may represent an additional useful biomarker for the assessment of the severity of cholelithiasis in dogs.

Three potential pathophysiological factors may affect the development of gallstones, including hypomotility of gallbladder, changes in bile properties due to dysregulated absorption/secretion, and hypersecretion of mucin. A growing number of studies showed that leptin levels may be associated with these abnormalities, either directly or indirectly. The contractile responses of gallbladder are triggered by neurotransmitters such as neuropeptide Y (NPY) and cholecystokinin (CCK), and both NPY and CCK have direct excitatory effects in the biliary tract through the stimulation of cholecystic contractility and the sphincter of Oddi [20, 34]. Previous research reported that leptin-deficient or leptin-resistant obese mice exhibited decreased gallbladder responses to NPY and CCK compared with control mice with normal leptin metabolism, suggesting that leptin dysregulation is associated with gallbladder motility and gallstone formation [20, 35]. The emptying of the postprandial gallbladder (up to 80% of the fasting gallbladder volume) is mostly controlled by CCK [36]. Leptin resistance decreases the sensitivity of vagal afferent neurons to CCK, thus inhibiting motility and emptying of the gallbladder [37]. One study [8] revealed leptin-regulated gallbladder-associated genes, such as aquaporin 1 water channel, aquaporin 4, epithelial sodium channel-α, and sodium hydrogen exchangers, which were shown to be related to absorption/secretion. Therefore, it was suggested that bile properties may be altered due to leptin dysregulation. Another study [19] reported an association between serum leptin and GBM suggesting that leptin imbalance is also related to the hypersecretion of mucus. We also demonstrated a relative increase in leptin and leptin receptor mRNA expression in the gallbladder tissues of dogs with cholelithiasis compared with that in healthy dogs. Based on these results, we can hypothesize that the upregulation of the expression of leptin and its receptor in the gallbladder of cholelithiasis patients is a response aimed at maintaining the homeostasis of gallbladder motility and bile composition under pathological conditions.

Hyperlipidemia represents an increased concentration of lipid fractions, such as total cholesterol and triglycerides in the blood [38]. The results of our study are consistent with those of previous studies in human patients, showing that the serum concentrations of total cholesterol and triglycerides in cholelithiasis patients were higher compared with those in the controls [21, 39]. In addition, hypercholesterolemia and hypertriglyceridemia were found to represent high risk factors for the development of cholelithiasis [40, 41]. The hypersecretion of cholesterol in the bile and the subsequent supersaturation of bile contribute to the formation of biliary sludge, which consequently reduces gallbladder motility [40]. The gallbladder motility of patients with hypertriglyceridemia is reduced compared to that of body mass index (BMI)-matched controls [41]. In a study investigating the association between gallbladder dysmotility and hypertriglyceridemia, decreased sensitivity to CCK, rather than reduced CCK release, was revealed to be the cause of impaired gallbladder motility in patients with high triglyceride blood levels [41].

In this study, we determined that both serum cholesterol and triglyceride levels positively correlate with leptin levels in both healthy and cholelithiasis patients. The association between the serum concentration of leptin and that of total cholesterol or triglycerides is controversial. In a study investigating leptin and cholesterol levels in lean (BCS 4-5/9) and obese (BCS 7-9/9) dogs, a positive correlation between leptin and cholesterol levels was reported [23]. The results of another study indicated the positive association between leptin and triglyceride levels in obese dogs as well [42]. In contrast, our previous GBM research revealed that, although many GBM patients had hyperlipidemia, no correlation between serum total cholesterol or triglyceride and leptin levels was detected [19]. One study showed that the associations between leptin and lipid [low-density lipoprotein (LDL), high-density lipoprotein (HDL), total cholesterol, and triglyceride] levels were not statistically significant in patients with combined hyperlipidemia [39]. Relationships between leptin levels and lipid profiles may differ according to the pathological conditions of the patient, such as GBM [19], obesity [23, 42], and hyperlipidemia [39].

In humans, previous studies investigating the associations between DM, insulin resistance, and leptin have been reported. Previously, it was suggested that leptin and insulin levels, and insulin resistance have different associations with obesity [43, 44]. DM is a well-known risk factor for gallstone formation [45]. In diabetic patients, cholesterol is hypersecreted during liver metabolism, leading to an increase in its absolute concentration in bile and causing a change in its relative proportion, which in turn affects the composition of the bile [46]. Insulin affects cholesterol saturation and gallbladder motility, an important factor in the formation of gallstones. In particular, hyperinsulinemia reduces gallbladder motility induced by CCK and increases the incidence of gallstones [47]. Hyperinsulinemia due to the insulin resistance affects the Na^+^-K^+^ pump activity, it regulates the release of neurotransmitters in presynaptic nerve terminals, and changes gallbladder smooth muscle tone. Therefore, it can lead to an increased gallbladder motility and decreased gallstone formation [48]. In one study in humans, serum leptin levels were shown to be directly related to insulin levels regardless of age and gender [49]. Other studies demonstrated that insulin resistance is most closely correlated with the leptin levels [50]. Taken together, these results suggest that leptin, insulin, and insulin resistance are associated with cholelithiasis. In our study, serum insulin levels were significantly higher in patients with cholelithiasis than those in the controls, but no statistically significant relationship between hyperinsulinemia and cholelithiasis was detected. Moreover, we showed that there were no significant differences in serum leptin and insulin concentrations between cholelithiasis patients with and without DM. These findings are consistent with previous investigations, showing that the leptin levels are associated with the disease status (chronic kidney disease, hyperadrenocorticism, and acute pancreatitis) [51] or obesity [52] rather than the presence of DM. Additionally, unlike the results showing a direct association between leptin and insulin levels [50], we observed no statistically significant associations between leptin and insulin concentrations in both the control and cholelithiasis group. Furthermore, only two of 34 dogs with cholelithiasis exhibited insulin resistance, and the number of dogs with hypercholesterolemia or hypertriglyceridemia of the total number of diabetic dogs was 1/7 and 2/7, respectively. Therefore, further studies on the associations between these factors are required.

This study has several limitations. First, we included a relatively small number of control and cholelithiasis patients. Analyses with more samples would provide more precise and reliable results. Due to the small number of samples, the associations between cholelithiasis, DM, and insulin resistance were not analyzed. Second, control over precise histories and analysis groups may be limited by the lack of complete medical records, including diagnostic and treatment histories. If this is the case, incorrect groupings may have influenced the results of the present study. If dogs had already begun receiving treatment for DM at the time of sample collection, this may have affected the observed differences in serum concentrations of leptin, insulin, and glucose between dogs with and without DM. Finally, our quantitative assessment of the relative expression differences of leptin and leptin receptor in the gallbladder tissues was performed using real-time PCR and not at the protein level.

In conclusion, in this study, we demonstrated that an increase in serum leptin concentrations and hyperlipidemia (hypercholesterolemia or hypertriglyceridemia) is associated with the occurrence of cholelithiasis in dogs, and the results of this study suggest that the homeostatic imbalance of these parameters may affect the pathogenesis of gallstones. The changes in the mRNA expression of leptin and leptin receptor within the gallbladder of dogs with cholelithiasis further underline that leptin may be a factor involved in the development of cholelithiasis, considering its important physiological role in gallbladder. However, further studies are required to definitively elucidate the relationship between hyperlipidemia and leptin in the pathophysiology of gallstones disease.
